# The TNF Paradox in Cancer Progression and Immunotherapy

**DOI:** 10.3389/fimmu.2019.01818

**Published:** 2019-07-31

**Authors:** Anne Montfort, Céline Colacios, Thierry Levade, Nathalie Andrieu-Abadie, Nicolas Meyer, Bruno Ségui

**Affiliations:** ^1^INSERM UMR 1037, Cancer Research Center of Toulouse (CRCT), Toulouse, France; ^2^Université Toulouse III - Paul Sabatier, Toulouse, France; ^3^Laboratoire de Biochimie, Institut Fédératif de Biologie, CHU Purpan, Toulouse, France; ^4^Dermatologie, Institut Universitaire du Cancer (IUCT-O) et CHU de Toulouse, Toulouse, France

**Keywords:** tumor necrosis factor (TNF), PD-1, CTLA-4, melanoma, immune escape

Tumor necrosis factor alpha (TNF)-dependent modulation of immune responses and cell death processes has long been the subject of intense research. Yet, its role in cancer progression is still a matter of debate. Here, we will (i) summarize key findings linking TNF to the promotion or inhibition of tumor progression, (ii) attempt to reconcile some of the contradictory findings, and (iii) describe the scientific rationale for improving the efficacy of immune checkpoint inhibitors *via* TNF blockade in metastatic melanoma patients.

## TNF as Anti-TUMOR Agent

**Figure 1:**
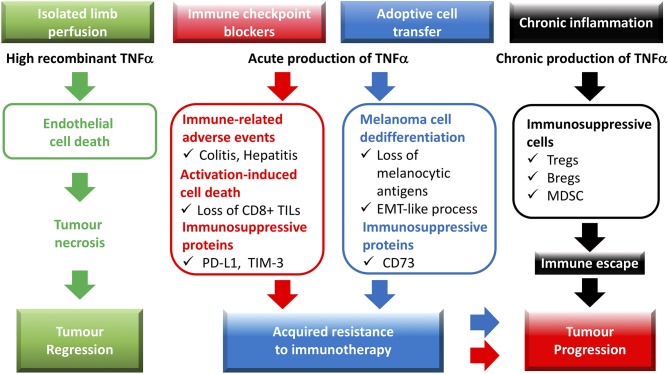
Role of TNF in cancer-associated immune responses: from tumor necrosis to resistance to immunotherapies and tumor progression. Recombinant TNF, administered by isolated limb perfusion, potently triggers endothelial cell death, and consequently, tumor necrosis. Immune checkpoint blockers promote an acute TNF production in the tumor microenvironment, which contributes to (i) the immune-related adverse events, (ii) the expression of the immunosuppressive molecules PD-L1 and TIM-3 on tumor-infiltrating leukocytes and/or cancer cells, (iii) the activation-induced cell death (AICD) process in CD8+ TILs. Adoptive T cell transfer of CD8 T cells is also associated with TNF production, which leads to melanoma dedifferentiation as well as expression of the CD73 ectonucleotidase. TNF-dependent expression of immunosuppressive molecules in the tumor microenvironment as well as AICD of CD8+ TILs and dedifferentiation of cancer cells favor acquired resistance to immunotherapies. During chronic inflammation, TNF likely contributes to immune escape and tumor progression by facilitating the biological activity and/or expansion of immunosuppressive cells such as regulatory T cells (Tregs), regulatory B cells (Bregs), and myeloid-derived suppressor cells (MDSCs).

Identified in 1975 and cloned in 1984, TNF was named regarding its capacity to induce the necrosis of transplanted methylcholanthrene-induced sarcomas in mice, when injected at a high concentration in tumors (1, 2). This phenomenon was later related to the ability TNF has to trigger apoptosis of tumor endothelial cells *via* ligation of the TNFR1 (3, 4). The high systemic toxicity associated with TNF treatment hampered the transposition of such a treatment to the clinic until two research teams developed procedures whereby high concentrations of TNF were perfused in isolated limbs of patients with melanoma or sarcoma (5, 6) (Figure 1). Building on these findings, studies focused on developing new strategies to augment TNF-mediated toxicity toward malignant cells. Notably, a fusion protein coupling TNF to the Cys-Asn-Gly-Arg-Cys peptide, able to target the aminopeptidase N expressed by tumor blood vessels, proved more efficient than TNF alone at reducing tumor growth in murine melanoma and lymphoma models (7), and promoted the efficacy of adoptive T cell transfer therapy (ACT) combined or not with anti-PD-1 treatments in mouse models of melanoma, and prostate carcinoma (8, 9). These results are in line with another work using a homotrimeric murine TNF molecule fused to a single-chain variable fragment (scFv) of the F8 antibody directed against the extra-domain A of fibronectin (10). Targeting this antigen, found in malignant tissues, allowed for TNF delivery in tumors, and favored the efficacy of peptide anticancer vaccine in mouse colon carcinoma. In another study, intra-tumor injection of an oncolytic adenovirus coding for murine TNF, and IL-2 also promoted anti-PD-1 efficacy in mouse melanoma (11). In these settings, improved CD8^+^ T cell infiltration in tumors was observed upon viral and anti-PD-1 combination therapy, although this phenotype is likely attributable to immune checkpoint blockade as viral therapy alone did not impact this parameter. Thus, delivering high concentrations of TNF in tumors is able to enhance the efficacy of immunotherapy. Of note, the direct impact of TNF on immune activation seemed often limited, and remains to be evaluated in more details. One might suggest however that, in this context, the TNF-induced tumor necrosis should promote some immune activation through the release of “danger signals,” and/or by increasing the delivery of antigens.

Interestingly, antigen-specific CD8^+^ T cells used TNF as part of their anti-tumor effector arsenal to kill MC38 mouse colon carcinoma cells (12). More precisely, production of TNF by perforin knockout OT-I CD8^+^ T cells, was toxic for ovalbumin-expressing MC38 cells (MC38^Ova^) in co-culture experiments. The authors showed that anti-PD-1 therapy slowed the progression of MC38^Ova^ tumors in perforin-deficient animals. This observation suggests that anti-PD-1 treatment could boost CD8^+^ tumor-infiltrating T lymphocytes (TILs) to use perforin/granzyme-independent cytotoxic mechanisms to impede cancer progression. However, direct implication of TNF in tumor growth control is lacking and would require evaluation of this phenomenon upon anti-PD-1/anti-TNF combination therapy. A recent study showed that tumor cell death following TNF production by CD4^+^ TILs during ACT required co-treatment with chemotherapeutic agents in murine models of lymphoma as well as colorectal and mammary carcinoma (13). In this work, both production of TNF by antigen-specific CD4^+^ T cells and chemotherapy were necessary to increase the oxidative stress in tumors hence promoting mouse survival. These findings suggest that even in a context whereby high numbers of antigen-specific T cells manage to reach the tumor, the levels of TNF produced might still be insufficient to kill malignant cells unless they are pre-sensitized to its cytotoxic properties.

Overall, the above-mentioned studies show that despite its potential to activate cell death processes, physiological intra-tumor TNF levels are likely insufficient to induce cancer regression in mice as well as in patients. Finding ways to increase production of this cytokine can potentiate the efficacy of immunotherapy, yet selective targeting of TNF in the tumor mass as well as management of the toxicity associated with such approaches remain a concern.

## TNF as pro-TUMOR Agent

The first observation directly linking TNF to tumor promotion came from Prof. F. Balkwill's laboratory. In the DMBA/TPA-induced mouse skin carcinogenesis model, TNF, TNFR1 as well as TNFR2-deficiency markedly reduced papilloma development (14, 15). These results might have seemed counter-intuitive at the time, however, a plethora of research now support them. One argument would be that as much as high TNF levels impede tumor growth, low levels of this cytokine, as observed in tumors, would on the contrary sustain cancer development. As a matter of fact, in the B16 mouse melanoma model, secretion of low levels of TNF by cancer cells promoted the infiltration of tumors with myeloid cells. These were shown to express endothelial markers, which promoted tumor vascularisation and progression (16). In a mouse model of ovarian carcinoma, TNFR1 expression on CD4^+^ T cells was found necessary for IL-17 secretion and myeloid cell recruitment in tumors, a phenomenon also associated with cancer progression (17). Moreover, our team showed that TNF production in mouse melanoma triggered TNF-R1-dependent activation-induced cell death (AICD) of CD8^+^ TILs. Blocking TNF/TNFR1 signaling *in vivo* using targeting antibodies was able to increase the proportion of melanoma-specific CD8^+^ T cells in the microenvironment and delayed tumor growth (18). Other studies implicated TNF in the activation, function, and/or differentiation of immune regulatory cells, including myeloid-derived suppressor cells (19) or regulatory T cells (20), most likely in a TNF-R2-dependent manner. Considering experiments performed in TNF-deficient animals, the main sources of TNF production in tumors are likely cells from the stroma rather than malignant cells. Interestingly, Donia et al. (21) showed that MHCII expression by patient-derived melanoma cells was associated with increased numbers of CD4^+^ TILs in tumors, which were able to produce TNF. Additionally, by performing adoptive cell transfer experiments, TNF production by B cells was able to support skin carcinomagenesis (22).

Another interesting notion is the implication of tumor-associated TNF in the dedifferentiation of cancer cells. Indeed, following ACT therapy whereby gp100-specific CD8^+^ T cells are injected in mouse melanoma tumors, TNF production induces dedifferentiation processes leading to loss of melanocytic markers, decreased immunogenicity, and tumor relapse (23), phenomena which were recently observed in a melanoma patient treated with ACT (24) (Figure 1). TNF involvement in epithelial-to-mesenchymal transition (EMT) was also described in other cancer models including breast, lung, and renal cell carcinoma (25–27). In melanoma, TNF-dependent dedifferentiation processes were also associated with increased expression of immune checkpoint molecules such as PD-L1, and CD73 (28, 29) (Figure 1). Whereas, PD-L1 increase likely depends on NF-κB activation, MAPK signaling pathway, through the c-Jun/AP-1 transcription factor complex, activates CD73 expression (28, 29).

In summary, not only can TNF inhibit anti-tumor immune responses *via* direct modulation of the activation, function, and survival of leukocytes during cancer progression but it can also alter the phenotype of cancer cells so that they become less visible to T cells, and express immune inhibitory molecules.

## Combining TNF Blockade to Immune Checkpoint Blockers to Treat Melanoma

Although melanomas represent only 1% of all skin cancers, they are responsible for the majority of skin cancer deaths. The use of immune checkpoint inhibitors (ICI) considerably improved the prognosis for metastatic melanoma patients with an overall survival of 58% at 3 years when treated with a combination of anti-PD-1 (Nivolumab), and anti-CTLA4 (Ipilimumab) (30). However, median progression-free survival is still only 11.5 months with nearly all patients experiencing mild to severe (grade 3/4) immune-related adverse events (irAE) (Figure 1). Interestingly, Infliximab, a first-generation chimeric TNF blocking antibody is currently being used in the clinic to treat some of the irAEs, mainly colitis, sometimes triggered by ICI (31). The impact anti-TNF antibodies have on anti-cancer immune responses in these settings are not known. A recent study indicates that 1% of patients with advanced melanoma treated by ICI develop severe colitis, which can be efficiently cured with one infliximab infusion in most of the patients, without affecting disease outcome (32). A clinical study evaluating the tolerability of infliximab in advanced cancer patients shows no dose-limiting toxic (DLT) effects and no evidence of disease acceleration in any patient. Moreover, 7 out of 41 patients experienced disease stabilization, including 1 metastatic melanoma patient (33). Other studies indicate the safety and tolerability of administering anti-TNF (etanercept or infliximab) in cancer patients affected with ovarian cancer (34), or renal cell carcinoma (35).

Following on these observations, we emphasized that rather than trying to increase TNF levels in tumors, blocking it may constitute a viable strategy to boost response to ICI. In an immunogenic mouse melanoma model, anti-PD-1 therapy allowed for the regression of 20% of tumors, whereas combining anti-PD-1, and anti-TNF induced the regression of 75% of them (36). This was associated with an increased percentage of CD8^+^ TILs likely related to reduced AICD. Under these settings, anti-PD-1 therapy promoted the expression of TIM-3, a secondary immune checkpoint molecule at the surface of CD8^+^ TILs as well as PD-L1 on dendritic cells, and TILs (Figure 1). This phenomenon was abrogated upon anti-PD-1 and anti-TNF co-treatment. Of note, although TNF blockade slightly reduced the production of IFNγ by CD8^+^ TILs, it did not impact that of granzyme B, suggesting that inhibiting TNF signals does not significantly alter the cytotoxic potential of CD8^+^ TILs.

These results represented the foundation for starting a phase 1b clinical trial (TICIMEL, NCT03293784), held at the Toulouse Oncopole (promoter: Claudius Regaud institute) funded by Bristol-Myers Squibb (BMS), and directed by Prof N. Meyer, oncodermatologist at the Toulouse cancer center. In TICIMEL, which started in December 2017, 30 patients with metastatic melanoma are for the first time simultaneously treated with TNF blocking antibodies (Infliximab or Certolizumab), and ICI (Nivolumab and Ipilimumab). The primary objective of TICIMEL is to assess the incidence of DLT, evaluated 12 weeks after treatment induction. The secondary objectives are to evaluate (i) the safety and tolerability, (ii) the progression-free survival, and (iii) the objective response rate. For the pharmacodynamic aspects, the systemic as well as the tumor-associated immune activation will be monitored before and during the treatment cycles.

In line with our preclinical findings, Melero, and co-workers recently published an interesting study in mouse colon cancer models, indicating that TNF blockers (anti-TNF or Etanercept) not only improves the anti-tumor effect of ICI (i.e., anti-PD-1 and anti-CTLA-4 combination) but also reduce irAEs (37). TNF blockers enhance the specific immune response, most likely by limiting the AICD of specific CD8 T cells, and neutralize the TNF-dependent colitis, and hepatitis. The benefits of TNF blockade were further documented using the B16-Ova mouse melanoma model, reinforcing our own observations, and further justifying our ongoing TICIMEL clinical trial in advanced melanoma patients.

To summarize, TNF implication in cancer development is rather complex with a balance between high, and low levels of TNF having opposing effects on tumor growth. Finding ways to exploit this balance has the potential to help promoting anti-tumor immune responses and improving the efficacy of existing anti-cancer therapies, especially immunotherapies.

## Author Contributions

AM wrote the paper. CC, TL, NA-A, NM, and BS edited the paper.

### Conflict of Interest Statement

NM has worked as an investigator and/or consultant and/or speaker for: BMS, MSD, Amgen, Roche, GSK, Novartis, Pierre Fabre. BS has worked as an investigator and consultant for BMS. The remaining authors declare that the research was conducted in the absence of any commercial or financial relationships that could be construed as a potential conflict of interest.

## References

[1] Carswell EA Old LJ Kassel RL Green S Fiore N Williamson B. An endotoxin-induced serum factor that causes necrosis of tumors. Proc Natl Acad Sci USA. (1975) 72:3666–70. 10.1073/pnas.72.9.3666 1103152 PMC433057

[2] Pennica D Nedwin GE Hayflick JS Seeburg PH Derynck R Palladino MA. Human tumour necrosis factor: precursor structure, expression and homology to lymphotoxin. Nature. (1984) 312:724–9. 10.1038/312724a0 6392892

[3] Robaye B Mosselmans R Fiers W Dumont JE Galand P. Tumor necrosis factor induces apoptosis (programmed cell death) in normal endothelial cells in vitro. Am J Pathol. (1991) 138:447–53. 1992769 PMC1886201

[4] Tartaglia LA Ayres TM Wong GH Goeddel DV. A novel domain within the 55 kd TNF receptor signals cell death. Cell. (1993) 74:845–53. 10.1016/0092-8674(93)90464-2 8397073

[5] Eggermont AM Schraffordt Koops H Klausner JM Kroon BB Schlag PM Liénard D. (1996). Isolated limb perfusion with tumor necrosis factor and melphalan for limb salvage in 186 patients with locally advanced soft tissue extremity sarcomas. the cumulative multicenter European experience. Ann Surg. 224, 756–64; discussion 764–55. 10.1097/00000658-199612000-00011 8968230 PMC1235474

[6] Fraker DL Alexander HR Andrich M Rosenberg SA. Treatment of patients with melanoma of the extremity using hyperthermic isolated limb perfusion with melphalan, tumor necrosis factor, and interferon gamma: results of a tumor necrosis factor dose-escalation study. J Clin Oncol. (1996) 14:479–89. 10.1200/JCO.1996.14.2.479 8636761

[7] Curnis F Sacchi A Borgna L Magni F Gasparri A Corti A. Enhancement of tumor necrosis factor alpha antitumor immunotherapeutic properties by targeted delivery to aminopeptidase N (CD13). Nat Biotechnol. (2000) 18:1185–90. 10.1038/81183 11062439

[8] Calcinotto A Grioni M Jachetti E Curnis F Mondino A Parmiani G. Targeting TNF-α to neoangiogenic vessels enhances lymphocyte infiltration in tumors and increases the therapeutic potential of immunotherapy. J Immunol. (2012) 188:2687–94. 10.4049/jimmunol.1101877 22323546

[9] Elia AR Grioni M Basso V Curnis F Freschi M Corti A. Targeting tumor vasculature with TNF leads effector T cells to the tumor and enhances therapeutic efficacy of immune checkpoint blockers in combination with adoptive cell therapy. Clin Cancer Res. (2018) 24:2171–81. 10.1158/1078-0432.CCR-17-2210 29490991

[10] Probst P Stringhini M Ritz D Fugmann T Neri D. Antibody-based delivery of TNF to the tumor neovasculature potentiates the therapeutic activity of a peptide anticancer vaccine. Clin Cancer Res. (2019) 25:698–709. 10.1158/1078-0432.CCR-18-1728 30327303 PMC6978140

[11] Cervera-Carrascon V Siurala M Santos JM Havunen R Tähtinen S Karell P. TNFa and IL-2 armed adenoviruses enable complete responses by anti-PD-1 checkpoint blockade. Oncoimmunology. (2018) 7:e1412902. 10.1080/2162402X.2017.1412902 29721366 PMC5927535

[12] Kearney CJ Vervoort SJ Hogg SJ Ramsbottom KM Freeman AJ Lalaoui N. Tumor immune evasion arises through loss of TNF sensitivity. Sci Immunol. (2018) 3:eaar3451. 10.1126/sciimmunol.aar3451 29776993

[13] Habtetsion T Ding ZC Pi W Li T Lu C Chen T. Alteration of tumor metabolism by CD4+ T cells leads to TNF-α-dependent intensification of oxidative stress and tumor cell death. Cell Metab. (2018) 28:228–42.e226. 10.1016/j.cmet.2018.05.012 29887396 PMC6082691

[14] Moore RJ Owens DM Stamp G Arnott C Burke F East N. Mice deficient in tumor necrosis factor-alpha are resistant to skin carcinogenesis. Nat Med. (1999) 5:828–31. 10.1038/10552 10395330

[15] Arnott CH Scott KA Moore RJ Robinson SC Thompson RG Balkwill FR. Expression of both TNF-alpha receptor subtypes is essential for optimal skin tumour development. Oncogene. (2004) 23:1902–10. 10.1038/sj.onc.1207317 14661063

[16] Li B Vincent A Cates J Brantley-Sieders DM Polk DB Young PP. Low levels of tumor necrosis factor alpha increase tumor growth by inducing an endothelial phenotype of monocytes recruited to the tumor site. Cancer Res. (2009) 69:338–48. 10.1158/0008-5472.CAN-08-1565 19118019 PMC2651676

[17] Charles KA Kulbe H Soper R Escorcio-Correia M Lawrence T Schultheis A. The tumor-promoting actions of TNF-alpha involve TNFR1 and IL-17 in ovarian cancer in mice and humans. J Clin Invest. (2009) 119:3011–23. 10.1172/JCI39065 19741298 PMC2752076

[18] Bertrand F Rochotte J Colacios C Montfort A Tilkin-Mariamé AF Touriol C. Blocking tumor necrosis factor α enhances CD8 T-cell-dependent immunity in experimental melanoma. Cancer Res. (2015) 75:2619–28. 10.1158/0008-5472.CAN-14-2524 25977337

[19] Zhao X Rong L Li X Liu X Deng J Wu H. TNF signaling drives myeloid-derived suppressor cell accumulation. J Clin Invest. (2012) 122:4094–104. 10.1172/JCI64115 23064360 PMC3484453

[20] Torrey H Butterworth J Mera T Okubo Y Wang L Baum D. Targeting TNFR2 with antagonistic antibodies inhibits proliferation of ovarian cancer cells and tumor-associated tregs. Sci Signal. (2017) 10:aaf8608. 10.1126/scisignal.aaf8608 28096513

[21] Donia M Andersen R Kjeldsen JW Fagone P Munir S Nicoletti F. Aberrant expression of MHC class II in melanoma attracts inflammatory tumor-specific CD4+ T- cells, which dampen CD8+ T-cell antitumor reactivity. Cancer Res. (2015) 75:3747–59. 10.1158/0008-5472.CAN-14-2956 26183926

[22] Schioppa T Moore R Thompson RG Rosser EC Kulbe H Nedospasov S. B regulatory cells and the tumor-promoting actions of TNF-α during squamous carcinogenesis. Proc Natl Acad Sci USA. (2011) 108:10662–7. 10.1073/pnas.1100994108 21670304 PMC3127875

[23] Landsberg J Kohlmeyer J Renn M Bald T Rogava M Cron M. Melanomas resist T-cell therapy through inflammation-induced reversible dedifferentiation. Nature. (2012) 490:412–6. 10.1038/nature11538 23051752

[24] Mehta A Kim YJ Robert L Tsoi J Comin-Anduix B Berent-Maoz B. Immunotherapy resistance by inflammation-induced dedifferentiation. Cancer Discov. (2018) 8:935–43. 10.1158/2159-8290.CD-17-1178 29899062 PMC6076867

[25] Ho MY Tang SJ Chuang MJ Cha TL Li JY Sun GH. TNF-α induces epithelial-mesenchymal transition of renal cell carcinoma cells via a GSK3β-dependent mechanism. Mol Cancer Res. (2012) 10:1109–19. 10.1158/1541-7786.MCR-12-0160 22707636

[26] Li CW Xia W Huo L Lim SO Wu Y Hsu JL. Epithelial-mesenchymal transition induced by TNF-α requires NF-κB-mediated transcriptional upregulation of Twist1. Cancer Res. (2012) 72:1290–300. 10.1158/0008-5472.CAN-11-3123 22253230 PMC3350107

[27] Liao SJ Luo J Li D Zhou YH Yan B Wei JJ. TGF-β1 and TNF-α synergistically induce epithelial to mesenchymal transition of breast cancer cells by enhancing TAK1 activation. J Cell Commun Signal. (2019). [Epub ahead of print]. 10.1007/s12079-019-00508-8 30739244 PMC6732143

[28] Reinhardt J Landsberg J Schmid-Burgk JL Ramis BB Bald T Glodde N. MAPK signaling and inflammation link melanoma phenotype switching to induction of CD73 during immunotherapy. Cancer Res. (2017) 77:4697–709. 10.1158/0008-5472.CAN-17-0395 28652246

[29] Zingg D Arenas-Ramirez N Sahin D Rosalia RA Antunes AT Haeusel J. The histone methyltransferase Ezh2 controls mechanisms of adaptive resistance to tumor immunotherapy. Cell Rep. (2017) 20:854–67. 10.1016/j.celrep.2017.07.007 28746871

[30] Wolchok JD Chiarion-Sileni V Gonzalez R Rutkowski P Grob JJ Cowey CL. Overall survival with combined nivolumab and ipilimumab in advanced melanoma. N Engl J Med. (2017) 377:1345–56. 10.1056/NEJMoa1709684 28889792 PMC5706778

[31] Draghi A Borch TH Radic HD Chamberlain CA Gokuldass A Svane IM. Differential effects of corticosteroids and anti-TNF on tumor-specific immune responses: implications for the management of irAEs. Int J Cancer. (2018) 145:1408–13. 10.1002/ijc.32080 30575963

[32] Lesage C Longvert C Prey S Maanaoui S Dreno B Machet L. Incidence and clinical impact of anti-TNFalpha treatment of severe immune checkpoint inhibitor-induced colitis in advanced melanoma: the mecolit survey. J Immunother. (2019) 42:175–9. 10.1097/CJI.0000000000000268 31090656

[33] Brown ER Charles KA Hoare SA Rye RL Jodrell DI Aird RE. A clinical study assessing the tolerability and biological effects of infliximab, a TNF-alpha inhibitor, in patients with advanced cancer. Ann Oncol. (2008) 19:1340–6. 10.1093/annonc/mdn054 18325912

[34] Madhusudan S Muthuramalingam SR Braybrooke JP Wilner S Kaur K Han C. Study of etanercept, a tumor necrosis factor-alpha inhibitor, in recurrent ovarian cancer. J Clin Oncol. (2005) 23:5950–9. 10.1200/JCO.2005.04.127 16135466

[35] Harrison ML Obermueller E Maisey NR Hoare S Edmonds K Li NF. Tumor necrosis factor alpha as a new target for renal cell carcinoma: two sequential phase II trials of infliximab at standard and high dose. J Clin Oncol. (2007) 25:4542–9. 10.1200/JCO.2007.11.2136 17925549

[36] Bertrand F Montfort A Marcheteau E Imbert C Gilhodes J Filleron T. TNFα blockade overcomes resistance to anti-PD-1 in experimental melanoma. Nat Commun. (2017) 8:2256. 10.1038/s41467-017-02358-7 29273790 PMC5741628

[37] Perez-Ruiz E Minute L Otano I Alvarez M Ochoa MC Belsue V. Prophylactic TNF blockade uncouples efficacy and toxicity in dual CTLA-4 and PD-1 immunotherapy. Nature. (2019) 569:428–32. 10.1038/s41586-019-1162-y 31043740

