# Corrigendum: The TNF Paradox in Cancer Progression and Immunotherapy

**DOI:** 10.3389/fimmu.2019.02515

**Published:** 2019-10-22

**Authors:** Anne Montfort, Céline Colacios, Thierry Levade, Nathalie Andrieu-Abadie, Nicolas Meyer, Bruno Ségui

**Affiliations:** ^1^INSERM UMR 1037, Cancer Research Center of Toulouse (CRCT), Toulouse, France; ^2^Université Toulouse III - Paul Sabatier, Toulouse, France; ^3^Laboratoire de Biochimie, Institut Fédératif de Biologie, CHU Purpan, Toulouse, France; ^4^Dermatologie, Institut Universitaire du Cancer (IUCT-O) et CHU de Toulouse, Toulouse, France

**Keywords:** tumor necrosis factor (TNF), PD-1, CTLA-4, melanoma, immune escape

In the original article, there was an error. We stated that Infliximab is “a first-generation humanized TNF blocking antibody,” instead of “a first-generation chimeric TNF blocking antibody.”

A correction has been made to the section **Combining TNF Blockade to Immune Checkpoint Blockers to Treat Melanoma**, paragraph one:

“Although melanomas represent only 1% of all skin cancers, they are responsible for the majority of skin cancer deaths. The use of immune checkpoint inhibitors (ICI) considerably improved the prognosis for metastatic melanoma patients with an overall survival of 58% at 3 years when treated with a combination of anti-PD-1 (Nivolumab), and anti-CTLA4 (Ipilimumab) (30). However, median progression-free survival is still only 11.5 months with nearly all patients experiencing mild to severe (grade 3/4) immune-related adverse events (irAE) (Figure 1). Interestingly, Infliximab, a first-generation chimeric TNF blocking antibody is currently being used in the clinic to treat some of the irAEs, mainly colitis, sometimes triggered by ICI (31). The impact anti-TNF antibodies have on anti-cancer immune responses in these settings are not known. A recent study indicates that 1% of patients with advanced melanoma treated by ICI develop severe colitis, which can be efficiently cured with one infliximab infusion in most of the patients, without affecting disease outcome (32). A clinical study evaluating the tolerability of infliximab in advanced cancer patients shows no dose-limiting toxic (DLT) effects and no evidence of disease acceleration in any patient. Moreover, 7 out of 41 patients experienced disease stabilization, including 1 metastatic melanoma patient (33). Other studies indicate the safety and tolerability of administering anti-TNF (etanercept or infliximab) in cancer patients affected with ovarian cancer (34), or renal cell carcinoma (35).”

The authors apologize for this error and state that this does not change the scientific conclusions of the article in any way. The original article has been updated.

